# Synthesis of Novel Reactive Disperse Silicon-Containing Dyes and Their Coloring Properties on Silicone Rubbers

**DOI:** 10.3390/molecules23010127

**Published:** 2018-01-09

**Authors:** Ning Yu, Shufen Zhang, Bingtao Tang, Wei Ma, Jinjing Qiu

**Affiliations:** 1 State Key Laboratory of Fine Chemicals, Dalian University of Technology, Dalian 116024, China; yuning@chemchina.com (N.Y.); tangbt@dlut.edu.cn (B.T.); weima@dlut.edu.cn (W.M.); 21207093@mail.dlut.edu.cn (J.Q.); 2 Department of Production & Operation, China National Chemical Corporation, Beijing 100080, China

**Keywords:** reactive disperse dyes, silicone rubber, coloring, silicone group

## Abstract

Novel red and purple reactive disperse silicon-containing dyes were designed and synthesized using *p*-nitroaniline and 6-bromo-2,4-dinitro-aniline as diazonium components, the first condensation product of cyanuric chloride and 3-(*N*,*N*-diethyl)amino-aniline as coupling component, and 3-aminopropylmethoxydimethylsilane, 3-aminopropylmethyldimethoxysilane, and 3-aminopropyltrimethoxysilane as silicone reactive agents. These dyes were characterized by UV-Vis, ^1^H-NMR, FT-IR, and MS. The obtained reactive disperse silicon-containing dyes were used to color silicone rubbers and the color fastness of the dyes were evaluated. The dry/wet rubbing and washing fastnesses of these dyes all reached 4–5 grade and the sublimation fastness was also above 4 grade, indicating outstanding performance in terms of color fastness. Such colored silicone rubbers showed bright and rich colors without affecting its static mechanical properties.

## 1. Introduction

Silicone rubbers have been extensively used in medical [1,2,3,4], aviation and aerospace fields [5,6,7], electric wires and cables [8,9,10,11], electronics and electric appliances [12,13], and automobiles and machines [14,15] due to their sound biosafety, high air permeability, excellent physical and mechanical properties, radiation-resistance, high temperature-resistance, low temperature flexibility, and weather resistance [16,17,18,19,20]. Many of their applications involve different colors of the silicone rubbers, and their common coloring technologies include internal coloring, external coloring, and color inserting [21]. Internal coloring is a process in which colorants are mixed with the silicone rubbers, making the color as the silicone rubbers’ base color. Internal coloring locks the colorant into the silicone rubbers, giving the color a “deepened” visual effect. However, mixing solid colorant into the silicone rubbers will compromise the mechanical property [22]. External coloring uses certain methods (such as spurting, applying, sprinkling or covering) to attach the colorant to the object and gives surfaces the target colors. However, the unavoidable contact of surfaces with the external environment will surely lead to fading or staining [23,24]. With regard to color inserting, a special device is used to insert the color into the silicone rubbers to give the color certain “deepened” effect. However, color inserting is a time- and cost-consuming process, and its many needle-pricking points will degrade the silicone rubbers’ physical and mechanical properties, especially at thin marginal areas. Therefore, color inserting is not frequently used.

**Scheme 1:**
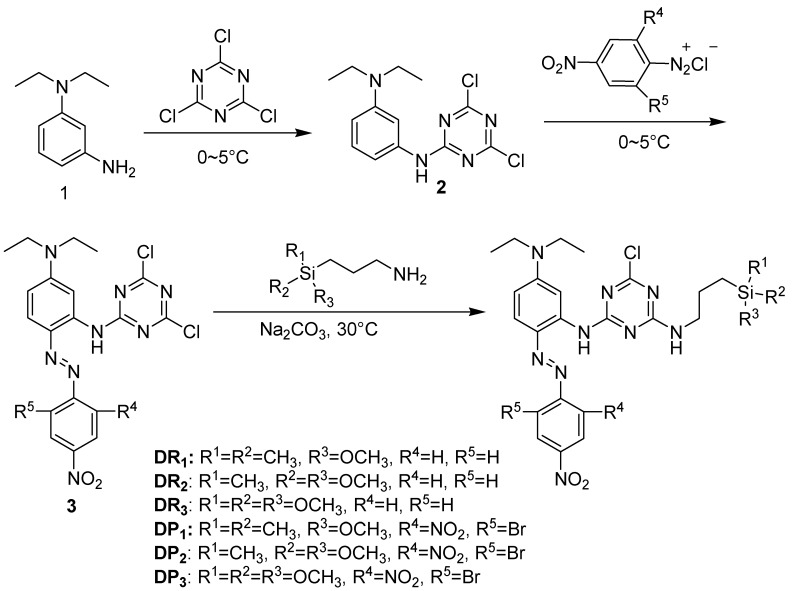
Synthesis of reactive disperse silicon-containing dyes.

The above-mentioned three common coloring technologies are all physical mixing between pigments and silicone rubbers. In this study, reactive disperse silicon-containing dyes were designed and synthesized for coloration of silicone rubbers through chemical bonding. Regarding azo dyes with multifunctional groups, post-modification strategies could be directly applied for further extension [25,26,27]. The dye molecular design idea of this article is that azo dyes and silicone groups are connected through linkage of cyanuric chloride. Thus, during coloration process, the reactive disperse silicon-containing dyes can covalently bond with silicone rubbers through Si-O bonding. The dyes were synthesized by a series of processes, including diazotization, coupling and condensing reactions, which introduced silane coupling agents of 3-aminopropylmethoxydimethylsilane, 3-aminopropylmethyldimethoxysilane, and 3-aminopropyltrimethoxysilane into the disperse dyes’ structure. The synthesis procedure of the reactive disperse silicon-containing dyes are shown in Scheme 1. UV-Vis absorption spectra, ^1^H-NMR, FT-IR spectra, and mass spectra were used for spectral and structural characterization of these dyes. After being colored with these novel dyes, the color fastness including washing, rubbing, and sublimation fastness of the dyes on silicone rubbers, as well as static mechanical properties of the colored rubbers were all measured. As the dyes were combined with the silicone rubbers through covalent bonds, stable and bright colors as well as decent mechanical properties of the silicone rubbers were achieved.

## 2. Experimental

### 2.1. Synthesis of Reactive Disperse Silicon-Containing Dyes

#### 2.1.1. Materials

All chemicals were reagent grade and did not undergo further purification. Distilled water was used for the experiments. Silicone rubber 1860 was supplied from Jiangxi Xinghuo Silicone Plant (Jiujiang, China). 3-(*N*,*N*-Diethyl)amino-acetylaniline (99%) was supplied by Shandong Xiya Corporation (Linyi, China). *p*-Nitroaniline (99%) was supplied by Adamas Reagent, Ltd. (Shanghai, China). 6-Bromo-2,4-dinitro-aniline (99%) was purchased from Shandong Changyi Zaohua Co., Ltd. (Weifang, China). Cyanuric chloride (99%), 3-aminopropylmethoxyldimethylsilane (97%), 3-aminopropylmethyldimethoxylsilane (97%), and 3-aminopropyltrimethoxylsilane (97%) were purchased from J&K Scientific Ltd. (Shanghai, China).

#### 2.1.2. Synthesis of Red Dye **DR_1_**

3-(*N*,*N*-Diethyl)amino-acetylaniline (11.00 g, 0.053 mol) was dissolved in ethanol (40 mL) at 20 °C and to this solution concentrated hydrochloric acid (20 mL) was added dropwise. The mixture was heated up to 70 °C and reacted for 10 h. The 3-(*N*,*N*-diethyl)amino-aniline (**1**) was obtained.

*p*-Nitroaniline (0.690 g, 5.0 mmol), 10 mL of water, and 3 mL of concentrated hydrochloric acid were stirred for 0.5 h at 75 °C. Then, the mixture was cooled to 0–5 °C and sodium nitrite (0.38 g, 5.5 mmol) was quickly added to obtain the corresponding *p*-nitroaniline diazonium salt solution. The course of the reaction was confirmed by thin-layer chromatography (petroleum ether:ethyl acetate, 4:1; *R*_f_ = 0.25). Raw material was fully converted after 20 min. 

Cyanuric chloride (3.68 g, 0.02 mol), toluene (10 mL), and 3-(*N*,*N*-diethyl)amino-aniline (**1**) (3.28 g, 0.02 mol) were mixed at 5 °C and stirred for 1 h. After that, the reaction solution was filtrated to give a gray filter cake of product **2**.

Intermediate **2** (1.550 g, 5 mmol) was dissolved in acetic acid (15 mL) and water (50 mL) at 5 °C. Concentrated hydrochloric acid (2 mL) was added to the above solution and then *p*-nitroaniline diazonium salt solution was added dropwise. The course of the reaction was confirmed by thin-layer chromatography (toluene:acetone, 6:1, *R*_f_ = 0.95). The solid **3** was obtained via neutralization to pH 7 with sodium hydroxide solution.

Product **3** (0.461 g, 1 mmol) and 0.18 mL of 3-aminopropyldimethylmethoxylsilane were then dissolved in tetrahydrofuran (60 mL). Then, the solution was warmed to 30 °C and stirred for 0.5 h. The course of the reaction was confirmed by thin-layer chromatography (toluene:acetone, 6:1; *R*_f_ = 0.75). The red solid was collected by filtration and dried to obtain 0.362 g of red dye **DR_1_** (yield 63%).

**DR_1_** purification was done by column chromatography (dichloromethane:ethyl acetate, 10:1; *v*/*v*).

The synthesis of red dye **DR_2_** (yield 61%) and **DR_3_** (yield 63%) was similar to the method used in the preparation of **DR_1_** (3-aminopropylmethyldimethoxylsilane and 3-aminopropyltrimethoxylsilane instead of 3-aminopropylmethoxyldimethylsilane were used, respectively).

#### 2.1.3. Synthesis of Purple Dye **DP_1_**

3-(*N*,*N*-Diethyl)amino-acetylaniline (11.00 g, 0.053 mol) was dissolved in ethanol (40 mL) at 20 °C. To this solution, concentrated hydrochloric acid (20 mL) was added dropwise, the mixture was heated up to 70 °C, and the reaction was continued for 10 h. 3-(*N*,*N*-Diethyl)amino-aniline (**1**) was obtained.

Sulfuric acid 98% (5 g, 0.05 mol) was added into sodium nitrite (0.38 g, 5.5 mmol) at room temperature, and stirred for 10 min. The mixture was cooled to 0–5 °C and 6-bromo-2,4-dinitro-aniline (1.31 g, 5.0 mmol) was added. The course of the reaction was confirmed by thin-layer chromatography (petroleum ether:ethyl acetate, 4:1; *R*_f_ = 0.25). Raw material was fully converted after 1 h. 6-Bromo-2,4-dinitro-aniline diazonium salt solution was obtained.

Cyanuric chloride (3.68 g, 0.02 mol), toluene (10 mL), and 3-(*N*,*N*-diethyl)amino-aniline (**1**) (3.28 g, 0.02 mol) were mixed at 5 °C and reacted for 1 h. After that, the reaction solution was filtrated to give gray filter cake of product **2**.

Intermediate **2** (1.550 g, 5 mmol) was dissolved in acetic acid (15 mL) and water (50 mL) at 5 °C. Concentrated hydrochloric acid (2 mL) was added to the above solution and then *p*-nitroaniline diazonium salt solution was added dropwise. The course of the reaction was confirmed by thin-layer chromatography (toluene:acetone, 6:1; *R*_f_ = 0.95). The solid **3** was obtained via neutralization to pH 7 with sodium hydroxide solution.

Product **3** (0.585 g, 1 mmol) and 0.18 mL of 3-aminopropylmethoxyldimethylsilane were dissolved in tetrahydrofuran (60 mL). Then, the solution was warmed to 30 °C and stirred for 0.5 h. The course of the reaction was confirmed by thin-layer chromatography (toluene:acetone, 6:1; *R*_f_ = 0.75). The purple solid was collected by filtration and dried to obtain 0.411 g of purple dye **DP_1_** (yield 59%).

**DP_1_** purification was done by column chromatography (dichloromethane:ethyl acetate, 10:1; *v*/*v*).

The synthesis of red dye **DP_2_** (yield 58%) and **DP_3_** (yield 57%) was similar to the method used in the preparation of **DP_1_** (3-aminopropylmethyldimethoxylsilane and 3-aminopropyltrimethoxylsilane instead of 3-aminopropylmethoxyldimethylsilane were used, respectively).

#### 2.1.4. Characterization

The UV-Vis absorption spectra of the corresponding dyes were measured by an Agilent 8453 UV-Vis spectrophotometer (Agilent, Santa Clara, CA, USA). ^1^H-NMR Spectra were recorded with a Varian INOVA 400 NMR spectrometer (Varian, Palo Alto, CA, USA). Infra-red spectra (20Dxb) were measured with Necolet (Madison, WI, USA). Mass spectra were recorded on CID (Collision-Induced Dissociation) = 50 V to 200 V with an HP 1100 high pressure liquid chromatography/mass spectrometry (HPLC/MS) system (1100 HPLC, Hewlett Packard, Palo Alto, CA, USA). 

#### 2.1.5. Structure Characterization Results of Reactive Disperse Silicon-Containing Dyes (Please see in Supplementary Materials)

Red dye **DR_1_**: C_25_H_34_ClN_9_O_3_Si: MS (API-ES) [M + H]^+^: 572. ^1^H-NMR data (ppm): (CDCl_3_, 400 MHz) δ: 0.07 (s, 6H), 0.56 (t, *J* = 6.6 Hz, 2H), 1.26 (t, *J* = 6.4 Hz, 6H), 1.85–1.86 (m, 2H), 3.01–3.02 (m, 2H), 3.49–3.50 (m, 4H), 3.44–3.45 (m, 3H), 5.32–5.33 (m, 2H), 6.39–6.41 (m, 1H), 7.75–7.86 (m, 3H), 8.16–8.29 (m, 3H). FT-IR (KBr, cm^−1^): 3407, 3273 (N-H); 1587, 1564, 1473 (benzene ring or cyanide ring); 1520, 1327 (NO_2_); 1261 (Si-Me); 1178, 1101, 1078 (Si-OMe). UV-Vis (nm): 512.1; molar absorptivity (L·mol^−1^·cm^−1^): 2.40 × 10^4^.

Red dye **DR_2_**: C_25_H_34_ClN_9_O_4_Si: MS (API-ES) [M + H]^+^: 588. ^1^H-NMR data (ppm): (CDCl_3_, 400 MHz) δ: 0.15 (s, 3H), 0.70 (t, *J* = 8.0 Hz, 2H), 1.30 (t, *J* = 6.8 Hz, 6H), 1.85–1.86 (m, 2H), 3.50–3.54 (m, 12H), 6.48–6.49 (m, 1H), 7.76–7.89 (m, 3H), 8.15–8.31 (m, 3H). FT-IR (KBr, cm^−1^): 3398, 3356, 3265 (N-H); 1562, 1473 (benzene ring or cyanide ring); 1516, 1325 (NO_2_); 1259 (Si-Me); 1176, 1101, 1076 (Si-OMe). UV-Vis (nm): 511.9; molar absorptivity (L·mol^−1^·cm^−1^): 2.41 × 10^4^.

Red dye **DR_3_**: C_25_H_34_ClN_9_O_5_Si: MS (API-ES) [M + H]^+^: 604. ^1^H-NMR data (ppm): (CDCl_3_, 400 MHz) δ: 0.72 (t, *J* = 8.0 Hz, 2H), 1.30 (t, *J* = 6.8 Hz, 6H), 1.77–1.78 (m, 2H), 3.51–3.60 (m, 15H), 6.44–6.46 (m, 1H), 7.76–7.87 (m, 3H), 8.15–8.29 (m, 3H). FT-IR (KBr, cm^−1^): 3396, 3373, 3267 (N-H); 1585, 1562, 1475 (benzene ring or cyanide ring); 1518, 1327 (NO_2_); 1178, 1101, 1078 (Si-OMe); 1176, 1101, 1076 (Si-OMe). UV-Vis (nm): 512.0; molar absorptivity (L·mol^−1^·cm^−1^): 2.40 × 10^4^.

Purple dye **DP_1_**: C_25_H_32_BrClN_10_O_5_Si: MS (API-ES) [M + H]^+^: 697. ^1^H-NMR data (ppm): (CDCl3, 600 MHz) δ: 0.14 (s, 6H), 0.68 (t, *J* = 6.6 Hz, 2H), 1.35 (t, *J* = 6.4 Hz, 6H), 1.68–1.70 (m, 2H), 3.47 (s, 3H), 3.47–3.48 (m, 2H), 3.57–3.60 (m, 4H), 6.51–6.53 (m, 2H), 6.99–6.70 (m, 1H), 8.39–8.39 (m, 1H), 8.66–8.66 (m, 1H). FT-IR (KBr, cm^−1^): 3417, 3361, 3269 (N-H); 1579, 1566, 1473 (benzene ring or cyanide ring); 1527, 1319 (NO_2_); 1259 (Si-Me); 1196, 1072 (Si-OMe). UV-Vis (nm): 553.5; molar absorptivity (L·mol^−1^·cm^−1^): 1.66 × 10^4^.

Purple dye **DP_2_**: C_25_H_32_BrClN_10_O_6_Si: MS (API-ES) [M + H]^+^: 713. ^1^H-NMR data (ppm): (DMSO-*d*_6_, 400 MHz) δ: 0.11 (s, 3H), 0.66 (t, *J* = 8.0 Hz, 2H), 1.30 (t, *J* = 6.8 Hz, 6H), 1.62–1.74 (m, 2H), 3.22 (s, 6H), 3.46–3.49 (m, 2H), 3.50–3.67 (m, 4H), 6.83–6.85 (m, 1H), 7.59–7.60 (m, 1H), 8.21–8.22 (m, 1H), 8.54 (s, 1H), 8.74–8.75 (m, 1H). FT-IR (KBr, cm^−1^): 3417, 3361, 3269 (N-H); 1566, 1473 (benzene ring or cyanide ring); 1527, 1319 (NO_2_); 1259 (Si-Me); 1196, 1072 (Si-OMe). UV-Vis (nm): 553.4; molar absorptivity (L·mol^−1^·cm^−1^): 1.66 × 10^4^.

Purple dye **DP_3_**: C_25_H_32_BrClN_10_O_7_Si: MS (API-ES) [M + H]^+^: 729. ^1^H-NMR data (ppm): (DMSO-*d*_6_, 400 MHz) δ: 0.48 (t, *J* = 8.0 Hz, 2H), 1.28 (t, *J* = 6.8 Hz, 6H), 1.65–1.67 (m, 2H), 3.22 (s, 9H), 3.50–3.63 (m, 6H), 6.77–6.79 (m, 1H), 7.52–7.53 (m, 1H), 8.16–8.18 (m, 1H), 8.48–8.50 (m, 1H), 8.72 (d, *J* = 7.2 Hz, 1H,). FT-IR (KBr, cm^−1^): 3407, 3363, 3269 (N-H); 1564, 1473 (benzene ring or cyanide ring); 1527, 1321 (NO_2_); 1196, 1074 (Si-OMe). UV-Vis (nm): 553.1; molar absorptivity (L·mol^−1^·cm^−1^): 1.66 × 10^4^.

### 2.2. Coloring the Silicone Rubbers

#### 2.2.1. Coloring Method

The dyes (0.1 g) were dissolved in 3 mL of tetrahydrofuran. Crude high-temperature vulcanized silicone rubber (HTVSR) (500.0 g) was processed on the open two-roll rubber mixing mill under room temperature. First, crude silicone rubber was added onto the roller, and then the mixing was started. The dye solution (10 mL), filling materials (1.0 g) (white carbon black made by sedimentation), and the platinum cross-linking agent (9.0 g) were gradually added. The compounding lasted for 20 min. The mixed silicone rubber was vulcanized on a compression molding press for 8 min at the temperature of 120 °C. All silicone rubbers were mold pressed and formed under the pressure of 10 MPa.

#### 2.2.2. Color Fastness Test

The rubbing fastness test was carried out according to the national standard GB/T 3920-2008; the sublimation fastness test according to GB/T 5718-1997; the washing fastness test according to GBT 3921-2008.

#### 2.2.3. Color Strength and Other Colorimetric Data Measurement

Color strength and other colorimetric data measurement were evaluated according to GBT 250-2008; color staining according to GBT 251-2008. 

The colored silicone rubber was used in acetic acid and ethanol; ammonia water (1%); DMF and water (*v*/*v*, 1:1); and DMF for stripping process. The silicon rubber was boiled in every solvent for 4 min. After boiled in one solvent, the silicon rubber was washed with temperature water. The color strength (K/S) and other colorimetric data of the colored silicone rubbers were measured using an UltraScan XE color Measuring and Matching Meter (HunterLab Co., Reston, VA, USA) at room temperature.

#### 2.2.4. Mechanical Property Test

The tensile strength, tensile stress at a given elongation and elongation at break tests were conducted according to GB/T 528-2009/ISO 37:2005; the tearing strength test according to GB/T 529-2008; the hardness test according to GB/T 531.1-2008/ISO 7619-1:2004; the elastic resilience test according to GB/T 1681-2009/ISO 4662:1986.

## 3. Results and Discussion

### 3.1. Dyes Synthesis and Structure Characterization

**Figure 1:**
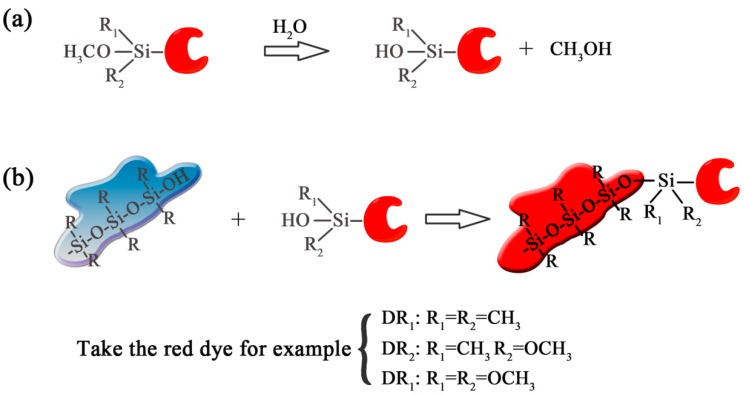
Illustration of coloring the silicone rubbers. (**a**) Formation of the dyes’ silanol; (**b**) Dyes’ silanol connected with the molecular of silicone rubbers through covalent bonds.

In order to design and synthesize a series of novel reactive disperse silicon-containing dyes (Figure 1), this paper used cyanuric chloride as linking group to bond azo disperse dye chromophore and silane coupling agent. Cyanuric chloride molecule has three active chlorine atoms. After one of the chlorine atoms was substituted by the amino group, the reactivity of the residual chlorines reduces due to the electron donation characteristic for amino group. Therefore, cyanuric chloride has temperature-controlled graded reactivity. In the 0–5 °C range, cyanuric chloride firstly reacted with 3-(*N*,*N*-diethyl)amino-aniline to give the coupling component **2**, which then coupled with arylamine diazonium salt affording the dye intermediate **3**. Finally, at 30 °C, the dye intermediate reacted with 3-aminopropylmethyldimethylsilane, 3-aminopropylmethyldimethoxysilane, and 3-aminopropyltrimethoxysilane, respectively, to give novel reactive disperse silicon-containing dyes.

The maximum absorption wavelength of **DR_1_**, **DR_2_**, and **DR_3_** was 512.1, 511.9, and 512.0 nm, respectively, and their molar absorptivity was 2.40 × 10^4^, 2.41 × 10^4^, and 2.40 × 10^4^ L·mol^−1^·cm^−1^, respectively. The maximum absorption wavelength of **DP_1_**, **DP_2_**, and **DP_3_** was 553.5, 553.4, and 553.1 nm, respectively, and their molar absorptivity was 1.66 × 10^4^, 1.66 × 10^4^, and 1.66 × 10^4^ L·mol^−1^·cm^−1^, respectively, thereby showing the strong color developing ability of the synthesized dyes. Mass spectra characterization of **DR_1_**, **DR_2_**, **DR_3_**, **DP_1_**, **DP_2_,** and **DP_3_** showed [M + H]^+^ at 572, 588, 604, 697, 713, and 729, respectively, thereby proving that the molecular weight of the synthesized dyes was correct. The characteristic infrared absorption showed the existence of the peaks of the secondary amine N-H stretching, benzene ring and triazine ring skeletal and nitro-group stretching, vibrations and characteristic Si-Me and Si-OMe peaks in all the molecules. ^1^H-NMR data indicated correct molecular structure of the synthesized dyes.

### 3.2. Color Fastness of the Colored Rubbers

The synthesized red silicon-containing reactive disperse dyes **DR_1_**, **DR_2_**, and **DR_3_** and purple silicon-containing reactive disperse dyes **DP_1_**, **DP_2_**, and **DP_3_** were used to color silicone rubbers, respectively. Color fastness values of the dyes in rubbers are shown in Table 1.

Table 1 shows that the dry/wet color fastness of red dyes **DR_1_**, **DR_2_**, and **DR_3_**, and purple dyes **DP_1_**, **DP_2_**, and **DP_3_** all reached 4–5 grade, their washing fastness also reached 4–5 grade and sublimation fastness was above 4 grade, demonstrating outstanding performance in all these three color fastness categories. This is due to the existence of the Si-O groups in the molecular structure of the novel silicon-containing dyes. In the vulcanization process, the siloxane group transformed to silanol (Figure 1a), which condensed with the silicone rubbers’ silanol and formed the Si-O-Si bonds, and further connected the dyes with the molecular structure of silicone rubbers through covalent bonds (Figure 1b), thus giving the colored silicone rubbers outstanding sublimation colorfastness.

### 3.3. Color Strength and Other Colorimetric Data of the Colored Rubbers

Silicone rubbers colored with red dyes **DR_1_**, **DR_2_**, and **DR_3_** and purple dyes **DP_1_**, **DP_2_**, and **DP_3_** received colorimetric measurement. The color strength and other color parameters L*, a*, b*, C*, and h* were compared. The colorimetric values of the colored rubbers are shown in Table 2.

The molar absorptivity of **DR_1_**, **DR_2_**, **DR_3_**, **DP_1_**, **DP_2_**, and **DP_3_** showed high color developing ability. Therefore, at the mass fraction of 0.02%, silicone rubbers colored with the red dyes **DR_1_**, **DR_2_**, and **DR_3_** and the purple dyes **DP_1_**, **DP_2_**, and **DP_3_** all recorded a K/S value are around 10 and 1:1 color depth (Table 2). Except that the brightness L*, color saturation C* and hue angle h* of red dyes **DR_2_** were slightly higher than those of **DR_1_** and **DR_3_**, the purple dyes **DP_1_**, **DP_2_**, and **DP_3_** had basically the same L*, C*, and h*, demonstrating a very close color shade of the dyes with the same chromophore. Moreover, chromaticity coordinate *x* and *y* of the dyes with the close color did not differ much.

### 3.4. Mechanical Properties of the Colored Rubber

Mechanical properties tests were carried out on rubbers colored with red dyes **DR_1_**, **DR_2_**, and **DR_3_**, and purple dyes **DP_1_**, **DP_2_**, and **DP_3_**, and changes to the parameters of the rubber such as tensile strength, tensile stress at a given elongation, elongation at break, tearing strength, hardness and elastic resilience were compared before and after the rubber was colored. The static mechanical properties of the rubbers colored with red and purple reactive disperse silicon-containing dyes are shown in Table 3 and Table 4, respectively.

Table 3 and Table 4 show that the colored silicone rubbers’ tensile strength largely ranged within 5.60–6.20 MPa, the tensile stress at a given elongation within 1.62–1.71 MPa, and the elongation at break within 348–396%, not changing much compared with those before being colored. This means coloring with these dyes did not affect the silicone rubbers’ elongation properties. Their hardness remained around 51, and the resilience remained around 50% without obvious changes, thereby indicating that the silicone rubbers’ elasticity was not affected as well. Their tearing strength obviously increased from 20.42 (before coloring) to 23.65–31.23 kN/m (after coloring), which was an increase of 15.8–52.9%, thereby demonstrating that these dyes gave the rubber not only outstanding color fastness but also excellent anti-tearing property. This result is due to the dyes being crosslinked with the silicone rubbers at the molecular level, which increased the silicone rubbers’ cross-linking degree and effectively reinforced its anti-tearing performance.

## 4. Conclusions

The novel red and purple reactive disperse silicon-containing dyes were designed and synthesized for the first time using 3-aminopropylmethoxydimethylsilane, 3-aminopropylmethyldimethoxysilane, and 3-aminopropyltrimethoxysilane as the silane coupling agents. These dyes were used for coloring the silicone rubbers and all the colored silicone rubbers’ dry/wet rubbing fastness and washing fastness reach 4–5 grade, and the sublimation fastness is also above 4 grade, outstanding results in terms of color fastness. Such colored silicone rubbers show bright and rich colors and decent mechanical properties. Therefore, the reactive disperse silicon-containing dyes used in silicone rubbers’ coloring would have good application in the future.

## Figures and Tables

**Table 1:** Color fastness properties of the silicon-containing reactive disperse dyes on rubbers.

Dyes	Washing			Rubbing		Sublimation		
	Color Change	Polyester	Cotton	Dry	Wet	Color Change	Polyester	Cotton
**DR_1_**	4–5	4–5	4–5	4–5	4–5	4–5	4–5	5
**DR_2_**	4–5	4–5	4–5	4–5	4–5	4	4–5	4–5
**DR_3_**	4–5	4–5	4–5	4–5	4–5	4	4–5	4–5
**DP_1_**	4–5	4–5	4–5	4–5	4–5	4	4–5	5
**DP_2_**	4–5	4–5	4–5	4–5	4–5	4–5	4–5	4–5
**DP_3_**	4–5	4–5	4–5	4–5	4–5	4–5	4–5	4–5

**Table 2:** Colorimetric data of the rubber colored with reactive disperse silicon-containing dyes.

	DR_1_	DR_2_	DR_3_	DP_1_	DP_2_	DP_3_
K/S	10.72	9.89	11.09	10.67	9.27	10.52
L*	37.6	42.87	37.15	30	32.25	30.6
C*	39.57	45.96	39.54	27.31	28.55	29.37
h*	12.93	14.45	11.05	314.14	314.43	315.77
a*	38.56	44.51	38.81	19.02	19.99	21.04
b*	8.85	11.47	7.58	−19.6	−20.39	−20.49
X	14.86	20.12	14.59	7.78	8.98	8.29
Y	9.87	13.07	9.62	6.24	7.19	6.48
Z	7.83	9.79	7.97	12.98	14.9	13.75
x	0.4565	0.4682	0.4533	0.2882	0.289	0.2906
y	0.303	0.3041	0.299	0.231	0.2315	0.2273
YI E313	104.82	114.26	102.06	−76.93	−75.67	−77.5
WI E313	−56.78	−64.7	−47.6	196.73	196.19	201.35

**Table 3:** Static mechanical properties of the rubbers colored with red silicon-containing reactive disperse dyes.

	Tensile Strength (MPa)	Elongation at Break (%)	100% Tensile Stress at a Given Elongation (MPa)	Tearing Strength (kN/m)	Hardness (Shore Hardness A)	Resilience (%)
Blank	6.09	381.50	1.68	20.42	51.3	50.0
**DR_1_**	5.60	348.67	1.74	30.37	52.0	53.3
**DR_2_**	6.03	396.78	1.65	28.15	51.9	51.2
**DR_3_**	6.12	372.15	1.67	31.23	52.2	50.9

**Table 4:** Static mechanical properties of rubbers colored with purple silicon-containing reactive disperse dyes.

	Tensile Strength (MPa)	Elongation at Break (%)	100% Tensile Stress at a Given Elongation (MPa)	Tearing Strength (kN/m)	Hardness (Shore Hardness A)	Resilience (%)
Blank	6.09	381.50	1.68	20.42	51.3	50.0
**DP_1_**	6.01	392.00	1.62	28.57	51.7	50.0
**DP_2_**	5.89	386.23	1.63	23.65	51.0	51.3
**DP_3_**	6.20	379.23	1.71	26.77	50.4	49.2

## Supplementary Materials

The supplementary materials are available online.
